# A Theoretical and Practical Treatise on Midwifery, Including the Diseases of Pregnancy and Parturition

**Published:** 1850-03

**Authors:** 


### BIBLIOGRAPHICAL NOTICES.

*A Theoretical and Practical Treatise on Midwifery, including the Diseases of Pregnancy and Parturition.* By P. Cazeaux, Adjunct Professor in the Faculty of Medicine of Paris, etc. etc., (adopted by the Royal Council of Public Instruction.) *Translated from the second French edition, with occasional notes and a copious Index,* by Robert P. Thomas, M. D., Member of the Philadelphia County Medical Society, late Demonstrator of Anatomy in the Franklin Medical College, etc., *with one hundred and seventeen illustrations.* 8vo. pp. 765. Philadelphia, Lindsay and Blakiston, 1850.

Considering the numerous excellent and comprehensive systems of Midwifery which have appeared in this country within a very short period, there would seem to be but little necessity for adding another to their number, and yet, the treatise before us comes to us with recommendations of so high a character as to warrant the translator and publisher in placing it within the reach of the great body of the American Medical Profession. Its adoption by the Royal Council of Public Instruction—the position and character of its author as a teacher of obstetrics—his opportunities for clinical experience, and the fact of the early demand in France for a second edition, present strong extrinsic recommendations of the work, which are fully sustained by its intrinsic merits.

Written expressly for "the use of students of medicine, and those of midwifery especially," its teachings are plain and explicit, presenting "a condensed summary of the leading principles established by the masters of the obstetric art," and such clear practical directions for the management of the pregnant, parturient, and puerperal states, as have been sanctioned by the most authoritative practitioners, and confirmed by the author's own experience. Collecting his materials from the writings of the entire body of antecedent writers, carefully testing their correctness and value by his own daily experience, and rejecting all such as were falsified by the numerous cases brought under his own immediate observation, he has formed out of them a body of doctrine, and a system of practical rules, which he illustrates and enforces in the clearest and most simple manner possible.

The first two parts of the Treatise present a very full and excellent account of the anatomy of the female organs of generation—the physiology of generation—the anatomical changes which occur during the progress of gestation—the diagnosis of pregnancy—the history of the fœtus, and the pathology of gestation. The reader will find in the second part a very accurate account of the modern doctrines of ovulation, and an excellent and intelligible history of the changes which the human ovum undergoes from the period of fecundation to the termination of utero-gestation. The changes that take place in the ovary and ovulum, both before and after fecundation, are illustrated by numerous engravings, exhibiting the successive stages the ovum passes through, from its primary appearance as a microscopic point, to the formation of the corpus luteum in the non-fecundated state, and to the development of the allantois, in that of conception. Every particular, in fact, connected with the maturation and perfection of the human ovum, upon which there is a general agreement among the leading physiologists of the day, has been very clearly described and pictured by M. Cazeaux.

Part the third is devoted to the history of labor in general, its causes and phenomena; the several presentations and positions; the management of the parturient and puerperal states, and the attentions required by the mother and child immediately after the termination of labor. The mechanism of labor is very ably defined; we agree with the translator, that the doctrines inculcated on this all important subject by M. Cazeaux are founded in truth. "The author has adopted the simple and beautiful classification of M. Nægèle, by which the description of the whole process of delivery is wonderfully simplified, its comprehension is rendered more intelligible, and the indications for manual or instrumental aid, in cases of difficulty or deformity, become more clear and determinate."

The positions and presentations of the fœtus, and the several stages of labor in each, are illustrated by very excellent wood cuts, without which the young student would scarcely be able to understand the verbal exposition, however clearly and explicitly given.

The fourth part of the Treatise treats of Dystocia, or preternatural and painful labors. It consists of two divisions, in the first of which are considered the causes of dystocia, or the circumstances that require the intervention of art. The author distributes all difficult labors into three groups: 1, where they are rendered difficult, impossible, or dangerous by a deficiency or an excess of action in the expulsive forces; 2, where any obstacle whatever exists which might oppose the easy expulsion of the fœtus; and 3, where the labor is complicated by any accident, sufficiently grave to compromise the health or life of either the mother or child.

The second division of part four treats of obstetrical operations. An attentive study of this portion of the work is not only recommended to the student of midwifery, but we are persuaded that the practitioner will derive from it practical precepts of a most valuable character. To know when manual interference is absolutely necessary, and to resort to it promptly when this decision has been formed, and to wait patiently in all cases in which there is good reason for believing that delivery will be safely effected by the powers of nature, without the necessity of manual or instrumental aid, is the characteristic of the well instructed and judicious obstetrician, and the rules and precautions laid down by M. Cazeaux, are well calculated to form this characteristic. While he points out the mischievous tendency of an unnecessary resort to manual and instrumental means for terminating the labor, he, at the same time, inculcates the importance of the early use of those means whenever absolutely required to ensure the safety of the mother or child.

The chapter on the induction of premature labor is deserving of an attentive perusal.

Part the fifth and last, treats of the delivery of the after-birth. The principles and practice laid down by the author, are sound and judicious. They differ, however, in nothing from those adopted and practiced by every experienced accoucheur.

No notice is taken in the work before us of the use of the recently introduced anæsthetic agents in labor. This is much to be regretted. It is a subject upon which it is all important that the student of midwifery should be correctly instructed. Of the advantages and dangers of the production of anæsthesia in the parturient female, he should be clearly informed, as well as of the results of experience, and the judgment formed of the practice by the authoritative masters of the obstetric art. We feel somewhat surprised that the translator has not supplied the omission of the author in a note or additional chapter.

The translation of Dr. Thomas, so far as we can judge, without having carefully compared it with the original, is tolerably well executed; it is occasionally, perhaps, too literal. The style is plain, and in general, sufficiently clear, though marred by occasional Gallicisms. The notes added by the translator, are few and unimportant.

In the present notice of the treatise of M. Cazeaux we have aimed at nothing further than a general expression of our views of the leading character of the work. In a more minute and critical examination of its several parts, we should unquestionably be obliged to dissent occasionally from the author; but not, we admit, from any leading principle or rule of practice laid down by him. In the correctness of all the more important of his teachings, we fully acquiesce, and can very conscientiously recommend the work to the medical student, as one that will prove to him a safe and valuable guide to a knowledge of obstetrics.

